# Nucleotide resolution profiling of m^3^C RNA modification by HAC-seq

**DOI:** 10.1093/nar/gkaa1186

**Published:** 2020-12-11

**Authors:** Jia Cui, Qi Liu, Erdem Sendinc, Yang Shi, Richard I Gregory

**Affiliations:** Stem Cell Program, Division of Hematology/Oncology, Boston Children's Hospital, Boston, MA 02115, USA; Department of Biological Chemistry and Molecular Pharmacology, Harvard Medical School, Boston, MA 02115, USA; Stem Cell Program, Division of Hematology/Oncology, Boston Children's Hospital, Boston, MA 02115, USA; Department of Biological Chemistry and Molecular Pharmacology, Harvard Medical School, Boston, MA 02115, USA; Division of Newborn Medicine and Epigenetics Program, Department of Medicine, Boston Children's Hospital, Boston, MA 02115, USA; Division of Newborn Medicine and Epigenetics Program, Department of Medicine, Boston Children's Hospital, Boston, MA 02115, USA; Ludwig Institute for Cancer Research, Oxford Branch, Oxford University, Oxford OX3 7DQ, UK; Stem Cell Program, Division of Hematology/Oncology, Boston Children's Hospital, Boston, MA 02115, USA; Department of Biological Chemistry and Molecular Pharmacology, Harvard Medical School, Boston, MA 02115, USA; Department of Pediatrics, Harvard Medical School, Boston, MA 02115, USA; Harvard Initiative for RNA Medicine, Boston, MA 02115, USA; Harvard Stem Cell Institute, Cambridge, MA 02138, USA

## Abstract

Cellular RNAs are subject to a myriad of different chemical modifications that play important roles in controlling RNA expression and function. Dysregulation of certain RNA modifications, the so-called ‘epitranscriptome’, contributes to human disease. One limitation in studying the functional, physiological, and pathological roles of the epitranscriptome is the availability of methods for the precise mapping of individual RNA modifications throughout the transcriptome. 3-Methylcytidine (m^3^C) modification of certain tRNAs is well established and was also recently detected in mRNA. However, methods for the specific mapping of m^3^C throughout the transcriptome are lacking. Here, we developed a m^3^C-specific technique, Hydrazine-Aniline Cleavage sequencing (HAC-seq), to profile the m^3^C methylome at single-nucleotide resolution. We applied HAC-seq to analyze ribosomal RNA (rRNA)-depleted total RNAs in human cells. We found that tRNAs are the predominant m^3^C-modified RNA species, with 17 m^3^C modification sites on 11 cytoplasmic and 2 mitochondrial tRNA isoacceptors in MCF7 cells. We found no evidence for m^3^C-modification of mRNA or other non-coding RNAs at comparable levels to tRNAs in these cells. HAC-seq provides a novel method for the unbiased, transcriptome-wide identification of m^3^C RNA modification at single-nucleotide resolution, and could be widely applied to reveal the m^3^C methylome in different cells and tissues.

## INTRODUCTION

Dynamic and reversible RNA modifications are increasingly recognized as an important layer of gene expression regulation at the post-transcriptional level. Functional analyses have shown that RNA modifications play fundamental roles in both normal cellular homeostasis, development, and disease (1,2). Transfer RNA (tRNA) is the most heavily modified RNA species in cells containing an average of 13 modifications per molecule (3). These modifications include for example, methylation, acetylation, pseudouridylation, hydroxylation, thiolation, adenine deamination, etc., and depending on the modification can occur on any of the four RNA bases and at characteristic positions within different subsets of tRNAs (4). Since tRNAs lie at the center of the ribosomal decoding machinery, the chemical modifications on tRNA affect mRNA decoding efficiency (5), translational fidelity (6), and proteome integrity (7). Numerous studies have directly linked alterations in different tRNA modifications to a variety of human diseases including neurodevelopmental disorders, cancer, diabetes, and mitochondrial-linked deficiencies (8). The recent and rapid advancement in technologies for detection of specific RNA modifications has revealed that several of these modifications are also present on certain messenger RNA (mRNA), non-coding RNA (ncRNA), and microRNA (miRNA) (9). m^6^A is the most abundant and well-characterized RNA modification on mRNA (9). The m^6^A machinery is involved in diverse molecular processes including mRNA translation (10,11), co-transcriptional regulation (12), and mRNA decay (13). Due to the critical role of m^6^A in controlling oncogene expression and its dysregulation in cancer cells and tumors, the m^6^A machinery has become a promising therapeutic target for anti-cancer drug development (14,15).

3-Methylcytidine (m^3^C) modification was first reported in rat tRNA-Ser in 1971 (16) and yeast tRNA-Thr in 1977 (17). Later studies showed that m^3^C is found at position 32 on the anticodon loop of tRNA-Ser, tRNA-Thr, and tRNA-Arg in *Saccharomyces cerevisiae* and other yeast (18–21). In human, m^3^C modification is not only present at the C32 position, but it is also found on the variable loop of some tRNA species (21,22). tRNA residue 32 is known to interact with residue 38 to stabilize the structure of the anti-codon loop, which is believed to play an important role in decoding accuracy (23,24). Furthermore, other modifications in the tRNA variable loop such as 7-methylguanosine (m^7^G) and 5-methylcytosine (m^5^C) are required for normal mRNA translation and cell function (5,25). Altogether this suggests an important role of m^3^C modification of tRNA in controlling tRNA stability and/or function, mRNA translation and protein expression.

The first methyltransferase (MTase) (TRM140) responsible for tRNA m^3^C modification was identified in *S. cerevisiae* (18,19). Recently, the role of mammalian homologs of m^3^C MTase has also been reported (26). In human there are four m^3^C MTase-like proteins – METTL2A, METTL2B, METTL6, and METTL8, while in mice there are three – METTL2, METTL6, and METTL8. All contain the conserved S-adenosyl methionine (SAM) binding domain. Xu *et al.* reported based on mass spectrometry-based (LC-MS/MS) analysis of isolated RNA fractions, that these four enzymes can be classified into two groups where METTL2A/2B/6 modify tRNAs while METTL8 modifies mRNA (26). However, the full repertoire of m^3^C-modified tRNAs, the particular subset of m^3^C-modified mRNAs, as well as the position of the modified cytidines within these transcripts is still unknown, and whether other ncRNA species (rRNA, miRNA, long-non coding RNA, etc.) contain m^3^C modification remains to be determined (27). In addition, two m^3^C demethylases have been recently reported where ALKBH3 demethylates tRNAs and ALKBH1 demethylates mRNA in human cell lines (28,29). This suggests that m^3^C modification can be regulated dynamically in mammalian cells. However the molecular role of RNA m^3^C marks in gene regulation, its biological function in mammalian cells, and its possible relevance to human disease are still poorly understood.

In order to better study the role of RNA m^3^C modification in normal cells and diseases, it is essential to have a reliable high-throughput method to site-specifically detect and quantify m^3^C RNA modification throughout the transcriptome. Unlike for certain other RNA modifications including m^6^A or m^7^G, due to lack of suitable anti-m^3^C antibodies, no antibody-based RNA immunoprecipitation and sequencing (RIP-seq) approaches have been successfully performed. Currently available approaches to detect and quantify RNA m^3^C levels can be classified into three types: The first approach is LC–MS/MS analysis. While this method is quantitative it cannot readily determine which sites are m^3^C-modified nor can it be used for the unbiased detection of the modified subset of RNA species within a bulk population. The second approach is the traditional primer extension assay. While this method can be helpful in identifying sites of modification, it can only be applied to candidate RNAs and this method is not high-throughput nor can it distinguish between m^3^C and other RNA modifications that disrupt base-pairing to terminate the reverse transcriptase. Another major limitation of this approach is that reverse transcription may also be arrested by RNA secondary structures and/or by other RNA modifications, both of which are very common on tRNAs. The third approach, is the next-generation sequencing based methods such as DM-tRNA-seq for mapping m^1^A, m^1^G, m^2,2^G and m^3^C RNA modifications (30), and AlkAniline-seq for mapping m^7^G and m^3^C RNA modifications (31). These methods are both capable of detecting different types of RNA modifications at single-nucleotide resolution throughout the transcriptome. However, neither of these methods is m^3^C-specific, and therefore are subject to confounding technical and analytical issues related to the specific detection of m^3^C modification. Therefore in this report we developed Hydrazine-Aniline Cleavage sequencing (HAC-seq), a chemically-based sequencing technique to unbiasedly map and quantify m^3^C methylomes on tRNA and other RNA species throughout the mammalian transcriptome at single-resolution.

## MATERIALS AND METHODS

### Cell culture

MCF7 cells were cultured in DMEM high glucose medium supplemented with 10% fetal bovine serum, 2.05 mM l-glutamine, 100 units per ml each of penicillin and streptomycin in a humidified atmosphere with 5% CO_2_ at 37°C.

### RNA extraction and isolation

Small RNAs (<200 nt) were purified using the miRVana miRNA Isolation Kit (Thermo Fisher Scientific) following by the manufacturer's protocol. For RiboMinus Total RNA isolation, total RNA was first extracted using Trizol (Thermo Fisher Scientific). 10 μg total RNA was then used for ribosomal RNA depletion by using the RiboMinus™ Eukaryote Kit for RNA-seq (Thermo Fisher Scientific) following the manufacturer's protocol. For individual tRNA isolation, 10 μg small RNA was first annealed with a mix of three 1 μM biotin-labeled probes covering the entire tRNA sequence in 50 μl of annealing buffer (10 mM Tris, 1 mM EDTA, 50 mM NaCl (pH 8.0)). The annealed RNA-probe mixture was purified using Dynabeads MyOne Streptavidin C1 magnetic beads (Thermo Fisher Scientific) following the manufacturer's protocol. RNA was released by boiling the beads in water at 95°C for 5 min and immediately cooled on ice. The isolated tRNA was further cleaned by using the Dynabeads MyOne Streptavidin C1 magnetic beads and collecting the supernatant. The following 5′ biotin-labeled probes were used: ValCAC1: 5′-AACCACTACACTACAGAAGC-3′; ValCAC2: 5′-CTTTCGCGTGTGAGGCGAAC-3′; ValCAC3: 5′-GCTTCTGCCCGGTTTCGAACC-3′; SerGCT1: 5′-CCACTCGGCCAGGCCTCTCC-3′; SerGCT2: 5′-ACAATGGATTAGCAGTCCAT-3′; SerGCT3: 5′-TCGAACCCAGGATCTCCTGT-3′. Final RNA concentrations were measured using Nanodrop. The RNA quality was confirmed using a A260/280 ratio of ∼2.0.

### HPLC-MS/MS analysis of RNA

250 ng to 500 ng RNA was digested with 100 U S1 nuclease (Thermo-Fisher # EN0321) at 37°C for 2 h and dephosphorylated with 1 U rSAP (NEB # M0371S) at 37°C for 1 h. The 100 μl samples were filtered with Millex-GV 0.22u filters (Millipore Sigma # SLGV033RS). 5–10 μl from each sample was injected into the Agilent 6470 Triple Quad LC/MS instrument with Agilent Zorbax Eclipse C18 reverse phase HPLC column. The samples were run at 500 μl/min flow rate in mobile phase buffer A (water with 0.1% Formic Acid) and 0–20% gradient of buffer B (acetonitrile with 0.1% formic acid). MRM transitions are measured for cytidine (244.1–112.1), 5-methylcytidine and 3-methylcytidine (m^5^C and m^3^C) (258.1–126.1), and adenosine (268.1–136.1). Standard compounds for m^5^C (Cayman Chemical #16111) and m^3^C (Cayman Chemical #21064) were run on HPLC/MS-MS to optimize HPLC method and determine retention times for each nucleoside. For LC/MS-MS data collection and analysis, Agilent Mass Hunter LC/MS Data Acquisition Version B.08.00 and Quantitative Analysis Version B.07.01 software was used.

### Recombinant protein purification

pET30a-AlkB-WT and pET30a-AlkB-D135S plasmids from Tao Pan (Addgene plasmid # 79050 and #79051) were transfected into One Shot^®^ BL21 Star™ (DE3) competent cells. A single transformant was cultured in LB medium containing antibiotic at 37°C overnight. Bacteria were inoculated and cultured in LB medium at 37°C until OD_600_ ∼0.5. Protein expression was induced by adding IPTG to a final concentration of 0.5 mM at 16°C overnight. Cells were harvested and lysed by sonication. After centrifugation, the cell lysates were collected. Recombinant His-tagged proteins were purified using Ni-NTA Agarose (Qiagen) following the manufacturer's protocol. AlkB-WT and AlkB-D135S proteins were checked by SDS-PAGE followed by colloidal blue staining and dialyzed into BC100 low salt buffer (20 mM Tris–HCl pH 7.8, 10% glycerol, 100 mM KCl).

### Demethylation reactions

For small RNA, 1 μg of isolated small RNA was demethylated in 100 μl of demethylation buffer containing 80 pmol of AlkB-WT, 160 pmol of AlkB-D135S, 5 μl of RNasin, and 50 μl of 2× demethylation buffer. For total RNA, 5 μg of total RNA was demethylated in 100 μl of demethylation buffer containing 160 pmol of AlkB-WT, 200 pmol of AlkB-D135S, 5 μl of RNasin, 50 μl of 2× demethylation buffer. The 2× demethylation buffer contains 0.6 M KCl, 4 mM MgCl_2_, 0.1 mM (NH_4_)_2_Fe(SO_4_)_2_.6H_2_O, 0.6 mM 2-ketoglutarate, 4 mM l-ascorbic acid, 0.1 mg/ml BSA, and 0.1 M MES, pH 5.0. The demethylation reaction was performed at room temperature in the dark for 2 h, and quenched with 5 mM EDTA. RNAs were then purified by acid phenol–chloroform.

### HAC northern blot

2 μg small RNA was treated with 25 μl of ice-cold hydrazine buffer (10% hydrazine, 3M NaCl) on ice for 10 min to 4 h. Then RNA was precipitated by adding 225 μl of H_2_O, 25 μl of 3M sodium acetate, pH 5.5, and 0.75 ml of 100% ethanol. After centrifugation and washes, the RNA pellet was resuspended in 100 μl cleavage buffer (H_2_O:glacial acid:aniline = 7:3:1) and incubated at room temperature in the dark for 2 h. RNA was then ethanal precipitated and dissolved in H_2_O. RNA concentration was measured by Nanodrop. 500 ng of small RNA were mixed with 2× TBE-Urea loading buffer (Bio-Rad), denatured at 95°C for 5 min, placed on ice before loading. RNA samples were then separated by 15% TBE–urea gels (Bio-Rad) at 200 V for 1 h. After electrophoresis, RNAs were transferred onto nylon membrane. Membranes were then UV-crosslinked, pre-hybridized, and blotted with p32-radioactive probes against the 3′ end of indicated tRNAs. The following probes were used: ArgCCT: 5′-CACCCCAGATGGGACTCGAA; LeuCAG: 5′-GTCAGGAGTGGGATTCGAAC-3′; SerCGT: 5′-TCGAACCCAGGATCTCCTGT-3′; ThrAGT: 5′-TCGAACCCAGGATCTCCTGT-3′; mt-ThrTGT: 5′-TGTCCTTGGAAAAAGGTTTTC-3′; ValCAC: 5′-GTTTCCGCCCGGTTTCGAACC-3′. Band intensities were quantified using ImageJ.

### HAC-seq

HAC-seq was performed with biological replicates using ribominus total RNA from MCF7 cells in three groups: (i) Ctrl: total RNA with demethylase treatment, without HAC; (ii) HAC: total RNA without demethylase treatment, with HAC; (iii) DM-HAC: total RNA with demethylase treatment, with HAC. 2 μg ribominus total RNA with or without demethylase treatment was fragmented by incubating in 10 μl of RNA fragmentation reagents (Thermo Fisher Scientific, AM8740) at 70°C for 10 min. The fragmentation reaction was stopped by adding 20 mM EDTA, pH8.0. After ethanol precipitation, RNA fragments were dephosphorylated with Antarctic phosphatase (NEB) and re-phosphorylated with T4 PNK (Thermo Fisher Scientific) following the manufacturer's protocols. After end repair, RNA was purified by using the Zymo RNA-Clean&Concentrator-5 (Zymo Research). Then hydrazine/aniline-induced cleavage (HAC) was performed for HAC and DM-HAC groups. Equal amounts of HAC and DM-HAC RNA samples were treated with 25 μl of ice-cold hydrazine buffer (10% hydrazine, 3M NaCl) on ice for 4 h. The reaction was stopped by ethanol precipitation. Next, RNA pellet was resuspended in 100 μl cleavage buffer (H_2_O:glacial acid:aniline = 7:3:1) and incubated at room temperature at dark for 2 h. RNA was then ethanal precipitated and dissolved in H_2_O. Before cDNA library preparation, HAC samples were demethylated by treating with 160 pmol of AlkB-WT and 200 pmol of AlkB-D135S in 100 μl of demethylation buffer at room temperature in dark for 2 h. RNAs were next purified by acid phenol–chloroform. Lastly, 100 ng of Ctrl, HAC, and DM-HAC RNA fragments were used for cDNA library construction by using the NEBNext Small RNA Library Prep Set (NEB) following the manufacturer's protocol. In the reverse transcription step, RNAs were incubated at 60°C for 1 h to increase the RT efficiency. cDNA libraries were sequenced with Illumina Nextseq 500. HAC-seq data analysis was performed following the m^7^G TRAC-seq protocol (5,32). Briefly, after quality control and mapping, we calculated the cleavage ratio of site*_i_* as the ratio of the number of reads starting at site*_i+1_* to the read depth of site*_i+1_*. Position *i* was considered as the m^3^C site if it met the following three criteria: (i) the cleavage ratio in HAC group is at least 0.5; (ii) there is at least a significant (*P*< 0.05) 2.5-fold increase in cleavage ratio after HAC treatment (HAC) compared with untreated control (Ctrl); (iii) there is at least a significant (*P*< 0.05) 2.5-fold decrease in cleavage ratio if the RNAs were demethylated before HAC (DM-HAC) compared with HAC. These threshold values can be set case by case depending on the experiment. The number of cleavage sites and the cleavage ratio plots were exported for presentation in GraphPad Prism. The 7-nt sequences (3-nt upstream and 3-nt downstream of the m^3^C sites) were used to generate the conserved m^3^C motif on WebLogo (33).

### Statistical analysis

Statistical analysis was performed in Microsoft Excel. Unpaired Student's *t*-test was used for all statistical analysis where *P< 0.05.

## RESULTS

### Identification of m^3^C tRNA modification via hydrazine/aniline-induced chemical cleavage

**Figure 1.**
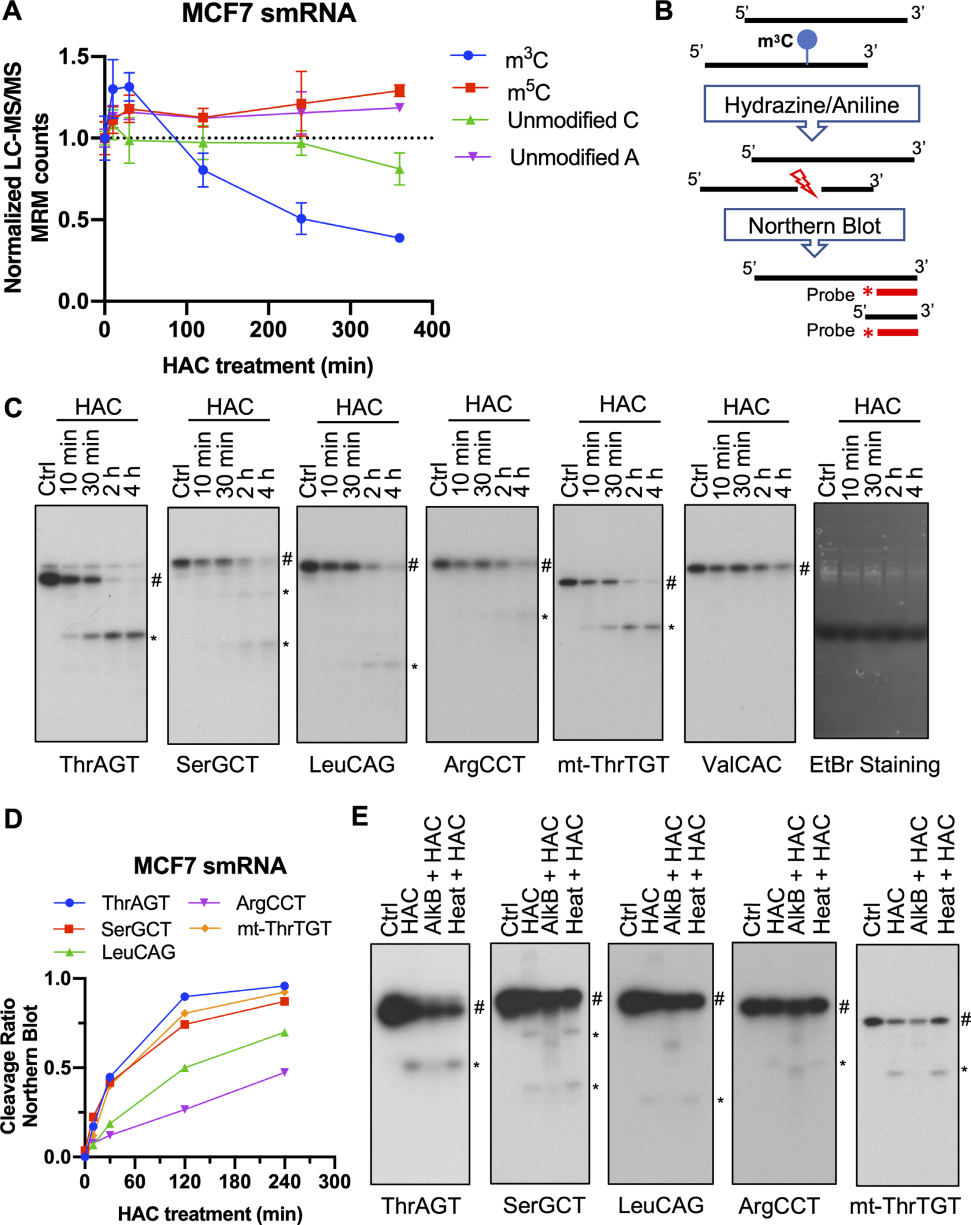
Use of hydrazine/aniline-induced chemical (HAC) cleavage for detection of m^3^C tRNA modification. (**A**) LC–MS/MS analysis of m^3^C, m^5^C, unmodified C and unmodified A on smRNA treated with hydrazine with different periods of time followed by aniline cleavage. Unmodified A was used as control. Data shown as mean ± SEM,*n* = 3. (**B**) Schematic of m^3^C detection using chemical cleavage of isolated small RNAs (smRNAs) and northern blot. (**C**) Representative northern blot analysis of hydrazine/aniline-treated small RNA samples. smRNA was treated with hydrazine with different periods of time followed by aniline cleavage. Five m^3^C-modified and one non- m^3^C-modified tRNAs were tested. 3′ cleaved fragments (*) were detected using indicated probes. EtBr staining is shown as a loading control. (**D**) Cleavage ratios were calculated by quantifying the band intensities of the cleaved (*) and uncleaved (#) bands in (C) for each m^3^C-modified tRNA. (**E**) Isolated small RNAs were untreated (Ctrl), treated with hydrazine/aniline (HAC), treated with AlkB before hydrazine/aniline treatment (AlkB + HAC), or heated before hydrazine/aniline treatment (Heat + HAC). Full-length (#) and 3′ cleaved fragments (*) were detected by northern blot.

Previous studies have shown that hydrazine can induce nucleophilic addition for m^3^C and unmodified U residues in RNA, but under high salt conditions (3M NaCl) the reaction is specific for m^3^C (34). After hydrazine treatment, subsequent aniline treatment could induce cleavage of the RNA chain at the m^3^C site (34). This chemical reaction was used in the late 1970s to study the sequence and structure of RNA under denaturing or native conditions (35,36). Based on this, we decided to make use of this m^3^C-specific chemical reaction to detect m^3^C on RNA. Since tRNAs are the mostly extensively studied cellular RNA species for m^3^C modification, they serve as the best positive controls to validate our method. We therefore developed our method using isolated small RNAs (smRNAs) (< 200 nt). We first incubated smRNAs (<200nt) with 10% hydrazine with 3M NaCl for different periods of time. Then RNAs were subjected to aniline-induced cleavage of the RNA chain. LC–MS/MS analysis showed that hydrazine/aniline treatment was able to decrease m^3^C levels on RNA in a time-dependent manner whereas it did not affect m^5^C or unmodified C levels (Figure 1A). In order to validate that hydrazine/aniline treatment could specifically cleave the RNA chain at m^3^C modification sites, the treated RNAs were further analyzed by Northern blot using specific RNA probes targeting the 3′ end of individual tRNAs (Figure 1B). We chose four known m^3^C-modified cytoplasmic tRNAs and one known m^3^C-modified mitochondrial tRNA as positive controls where ThrAGT, ArgCCT, mt-ThrTGT are modified at C32, LeuCAG is modified at C47d, and SerGCT is modified at both C32 and C47d. Our chemically-induced cleavage method was able to detect one or two cleaved 3′ fragments of the correct sizes (Figure 1C). The band intensities of the cleaved and the full-length (uncleaved) bands were further quantified and the cleavage ratio was calculated as the ratio of band intensities of the cleaved band to the sum of uncleaved and cleaved bands for each m^3^C-modified tRNA (Figure 1D). Consistent with our LC–MS/MS analysis, hydrazine/aniline treatment cleaved m^3^C-modified tRNAs in a time-dependent manner (Figure 1D). In order to verify that the chemically-induced cleavage we observed in Figure 1C was due to the known m^3^C modification at these sites, we performed two additional control experiments in Figure 1D. First, we treated small RNAs with bacterial AlkB to demethylate m^3^C as well as several other types of methylation before the hydrazine/aniline treatment. Second, we denatured small RNAs by heat before hydrazine/aniline treatment. As expected, the 3′ cleaved fragments were undetectable if small RNAs were demethylated before chemical treatment. Also, since the 3′ cleaved fragments were readily detectable in the heat denatured RNA samples we conclude that the chemical cleavage is unaffected by RNA structure. Therefore, we demonstrate that hydrazine/aniline treatment can specifically cleave tRNAs at m^3^C modification sites.

### Hydrazine–aniline cleavage sequencing (HAC-seq) experimental design

**Figure 2.**
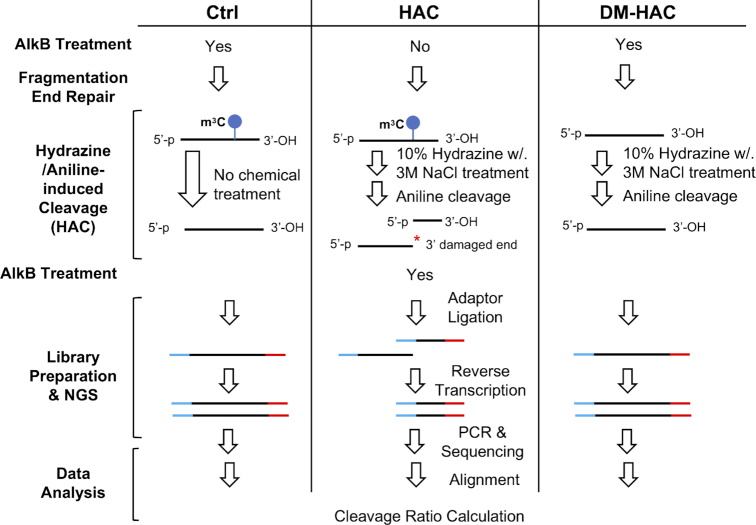
HAC-seq workflow. After ribosomal RNA (rRNA) depletion, RiboMinus total RNAs were randomly fragmented and end-repaired. The fragmented RNAs were treated with 10% hydrazine with 3M NaCl followed by aniline to induce the cleavage of the RNA backbone at the m^3^C modification sites. Then RNAs were treated with bacterial AlkB to remove the major types of modifications on tRNA. Two controls were performed at the same time. Untreated control (Ctrl) was carried out with AlkB demethylation but without HAC treatment. DM-HAC was carried out by AlkB demethylation to remove m^3^C before HAC-induced cleavage. The 5′ fragment generated by HAC contains a damaged 3′ end without the correct 3′-OH group which prevents the adaptor ligation in the library preparation step. Only the full length and 3′ cleaved fragments can be subsequently sequenced. After the bioinformatic data analysis, m^3^C-modified sites are determined by calculating the cleavage ratio at single nucleotide resolution.

In order to explore the global m^3^C RNA methylome we then coupled this hydrazine/aniline-induced chemical cleavage method with next generation sequencing to establish a hydrazine–aniline cleavage sequencing (HAC-seq) technique for the identification of m^3^C modification sites on RNAs at single-nucleotide resolution (Figure 2). Our LC–MS/MS analysis (Figure 1A) and northern blot analysis (Figure 1C and D) showed that 4 h hydrazine treatment followed by aniline cleavage reached the maximum decrease in global m^3^C levels and maximum cleavage ratio for individual m^3^C-modified tRNAs, respectively. HAC-seq was therefore performed using these same conditions. Total RNA was used to map the m^3^C methylome on all RNA species throughout the transcriptome. In order to get a better coverage for longer RNA species, after ribosomal RNA (rRNA) depletion, we fragmented (∼200 nt) these ribominus RNAs. We then repaired the 5′ and 3′ ends to make sure that the fragments contain the correct 5′ phosphate and 3′ OH groups. Fragmented RNAs were first treated with 10% hydrazine with 3M NaCl and then with aniline to induce the site-specific chemical cleavage. In order to get more sequence reads, we included a demethylation step (recombinant bacterial AlkB treatment) to remove other major types of methylation on small RNAs that can interfere with the reverse transcriptase production of cDNA. To validate that the cleavage sites detected by HAC-seq were due to methylation, total RNAs were pre-treated with demethylase AlkB to remove m^3^C on RNA before HAC treatment. Then cDNA libraries were prepared from untreated control RNA (Ctrl), HAC-treated RNA (HAC), and demethylated plus HAC-treated RNA (DM-HAC) samples with adaptor ligation and reverse transcription followed by PCR. If an RNA is m^3^C-modified, the hydrazine/aniline treatment cleaved the RNA and generated two or more cleaved fragments. The 3′ cleaved fragments contain the correct 5′ phosphate and 3′ OH groups for adaptor ligation for cDNA library construction. However the 5′ cleaved fragments have a 3′ damaged end which cannot be ligated with adaptors. Based on this, during the cDNA library preparation step only the 3′ cleaved fragments and the full-length RNAs could be sequenced. The sequence reads were aligned with reference RNA datasets to identify all chemically-induced cleavage sites. We next calculated the cleavage ratio at each nucleotide (*i*) throughout a RNA by dividing the number of reads starting at the following nucleotide (*i*+ 1) by the read depth at the following nucleotide (*i*+ 1) in the Ctrl, HAC and DM-HAC groups. m^3^C modification sites were determined by the following three criteria: (i) the absolute cleavage ratio in the HAC group was at least 50%; (ii) there was a significant 2.5-fold increase in cleavage ratio when comparing HAC with Ctrl; (iii) there was a significant 2.5-fold decrease in cleavage ratio when comparing DM-HAC with HAC since m^3^C was removed by AlkB before HAC treatment.

### Single-nucleotide resolution mapping of m^3^C modification of tRNA by HAC-seq

**Figure 3.**
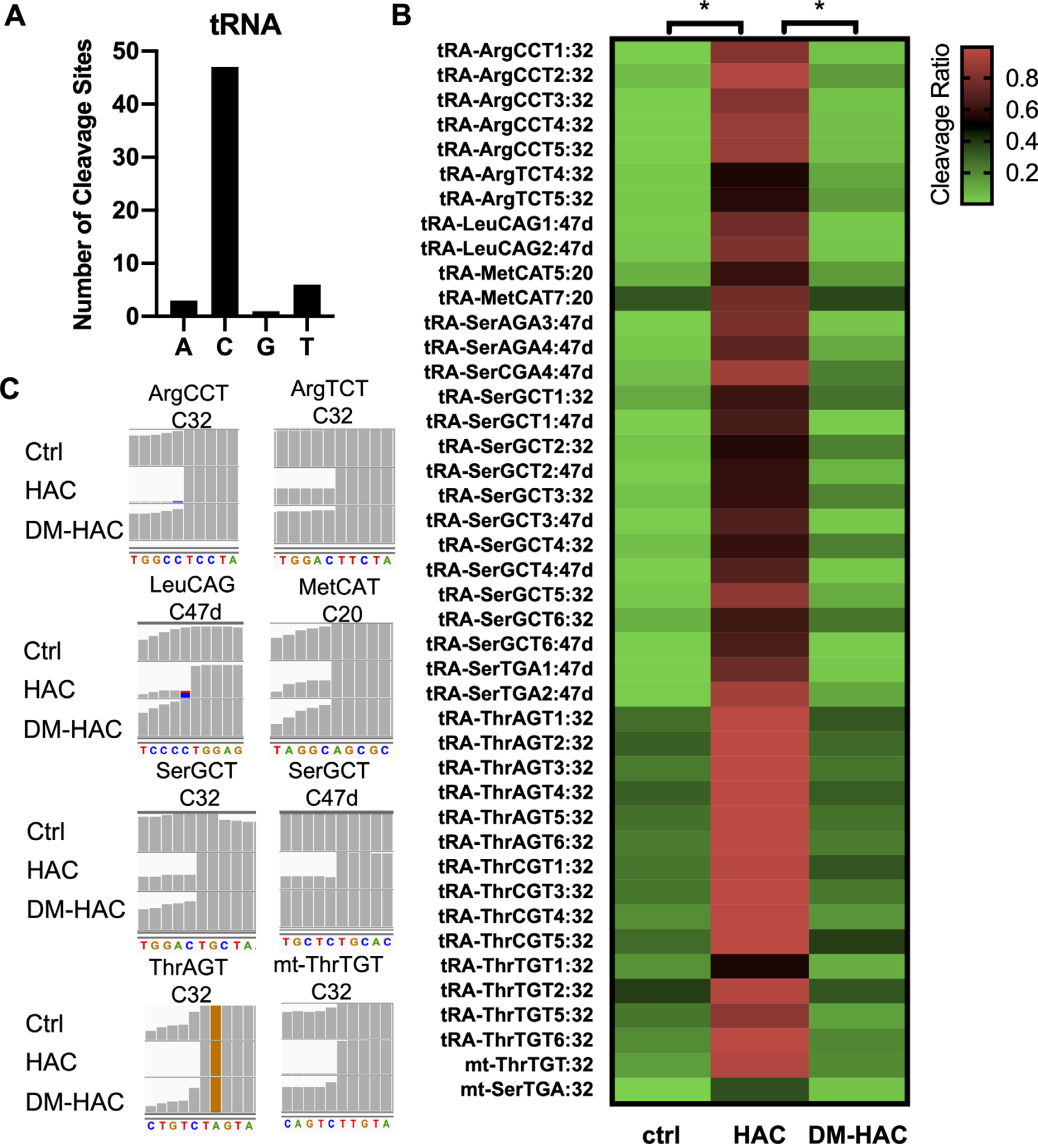
HAC-seq mapping of m^3^C tRNA modification sites at single nucleotide resolution. (**A**) Number of cleavage sites detected at nucleotides A, C, G and T by following the HAC-seq data analysis pipeline. (**B**) Heat map showing the cleavage ratios of all known m^3^C-modified tRNA sites calculated from HAC-seq datasets. Data was shown as the average cleavage ratios of two sequencing replicates using Ctrl, HAC, or DM-HAC RNA samples where *P< 0.05. (**C**) Representative read alignments of Ctrl, HAC and DM-HAC tRNAs from HAC-seq are shown in the Integrative Genomics Viewer (IGV). All m^3^C-modified tRNAs are indicated with a significant site-specific decrease in sequence reads upon HAC compared with Ctrl, which returned to Ctrl level in DM-HAC.

We first analyzed the m^3^C sites on tRNA by mapping the HAC-seq data to tRNA dataset. Our analysis revealed that C was the main nucleotide identified by HAC-seq as expected (Figure 3A). Together with our LC–MS/MS and Northern blot analysis in Figure 1, this indicates that HAC-seq is specific for detection of m^3^C sites on RNA. The cleavage ratios of the known m^3^C-modified tRNAs were calculated from HAC-seq using Ctrl, HAC and DM-HAC RNA samples and were further plotted as a heat map (Figure 3B). Representative IGV images of read alignments for individual m^3^C-modified tRNA isoforms showed significant decrease in sequence reads at m^3^C-modified site (Figure 3C). Removal of m^3^C by demethylase treatment (DM-HAC) prevented the chemical-induced cleavages (Figure 3B and C).

**Figure 4.**
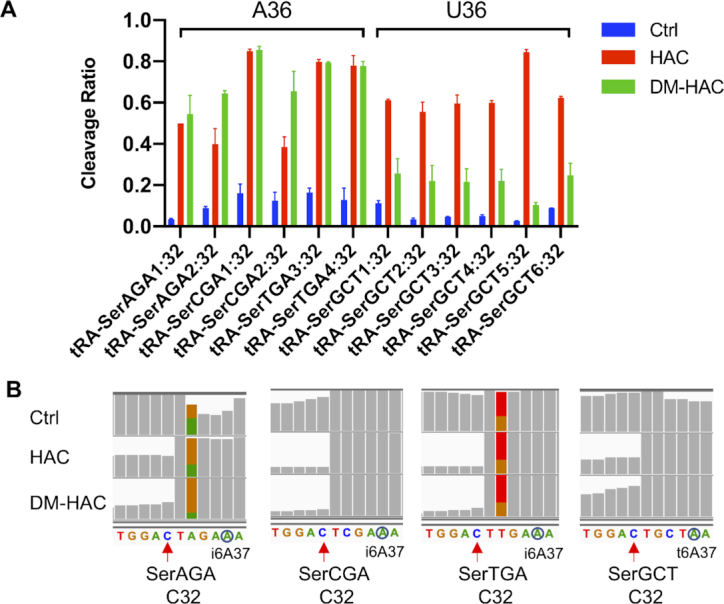
Differential detection of m^3^C32 modification of tRNA-Ser with A36 versus tRNA-Ser with U36 by HAC-seq. (**A**) Cleavage ratio plots of all m^3^C32 sites on different tRNA-Ser isoforms detected by HAC-seq. (**B**) IGV displays of read alignments around m^3^C32 sites on different tRNA-Ser isoforms. i^6^A36 and t^6^A36 are highlighted in blue circles.

We found that HAC-seq successfully detected both m^3^C32 and m^3^C47d in tRNA-SerGCT, but surprisingly only m^3^C47d sites were identified in tRNA-SerAGA, tRNA-SerCGA, and tRNA-SerTGA and not the expected m^3^C32 (Figure 3). This is because although HAC cleaved m^3^C32 in tRNA-SerAGA, tRNA-SerCGA and tRNA-SerTGA, the cleavage was not diminished by pre-treatment with AlkB (Figure 4). Therefore based on our criteria for calling m^3^C-positive sites, these positions were not considered as m^3^C. This indicates that m^3^C32 on tRNA-Ser species with A36 (SerAGA, SerCGA and SerTGA) is less sensitive to the AlkB treatment than those on tRNA-Ser species with U36 (SerGCT). tRNA-Ser that decodes codons beginning with U are different from tRNA-Ser that decodes with codons that begin with A in many ways. Previous research reported that tRNA-Ser with A36 showed lower levels of m^3^C compared with tRNA-Ser with U36 in *S. pombe* (21). Additionally, tRNA-Ser with A36 contain i^6^A37 while tRNA-Ser with U36 contain t^6^A37. i^6^A37 and t^6^A37 have been revealed to play an important role in stimulating m^3^C modification on tRNA-Ser species in *S. pombe* and *S. cerevisiae* (20,21). Structural studies showed that i^6^A37 modification destabilizes while t^6^A37 modification stabilizes the anticodon stem loop structure of tRNA (37,38). Since catalytic activity of the AlkB family proteins is highly affected by RNA structure (39), it is likely that i^6^A37 and t^6^A37 modifications on different tRNA-Ser species induce different structural conformation of the tRNA anticodon stem loop, which then affects the demethylation activity of AlkB.

**Figure 5.**
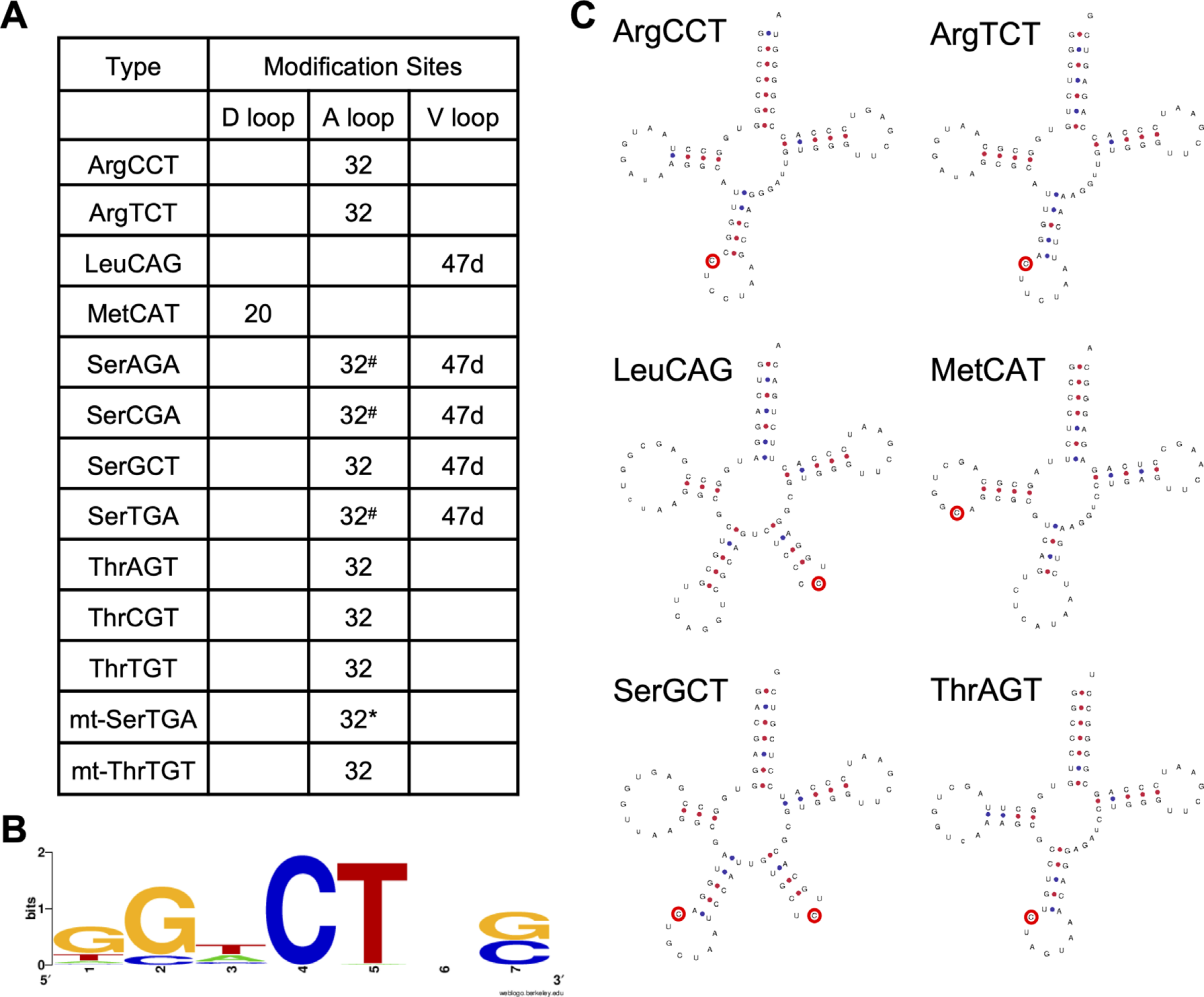
Summary of m^3^C-modified tRNAs identified by HAC-seq. (**A**) List of all m^3^C-modified tRNAs identified by HAC-seq analysis. ^#^Cleavages at m^3^C32 on tRNA-SerAGA, SerCGA, SerTGA are detected even if RNA is demethylated with AlkB before HAC. *Cleavage ratio of mt-SerTGA-C32 is below 0.5. (**B**) Conserved sequence motif identified in the canonical m^3^C-modified tRNAs using WebLogo. (**C**) Representative cloverleaf structures of m^3^C-modified tRNAs in (A) were shown. m^3^C modification sites were highlighted in red circles. All tRNA cloverleaf structures were downloaded from GtRNAdb.

Compared with the tRNA m^3^C methylome characterized by the non-m^3^C specific sequencing methods (22,30,31,40,41), all the reported tRNA m^3^C sites were detected by HAC-seq except for mitochondrial tRNA SerTGA (mt-SerTGA) m^3^C32 (Figure 5A). This is because the expression level of mt-SerTGA in MCF7 cells is low. Additionally, the cleavage ratio calculated from our HAC-seq data is around 35% which is below our cutoff criteria of 50% (Figure 3B). However our HAC-seq did detect significant cleavage at C32 in HAC, but not in the Ctrl or DM-HAC samples (Figure 3B). m^3^C sites occur within a conserved sequence motif (Figure 5B). HAC-seq also identified some sites that were not previously reported as m^3^C modified including (LeuCAG C50, ValAAC C50 and ValCAC C50) (Supplementary Figure S1A). However LC-MS/MS analysis (Supplementary Figure S1B) did not confirm m^3^C on these tRNAs. These false-positive sites are located in regions with relatively low read coverage, suggesting these apparent cleavages could have resulted from background sequencing noise (Supplementary Figure S1C).

**Figure 6.**
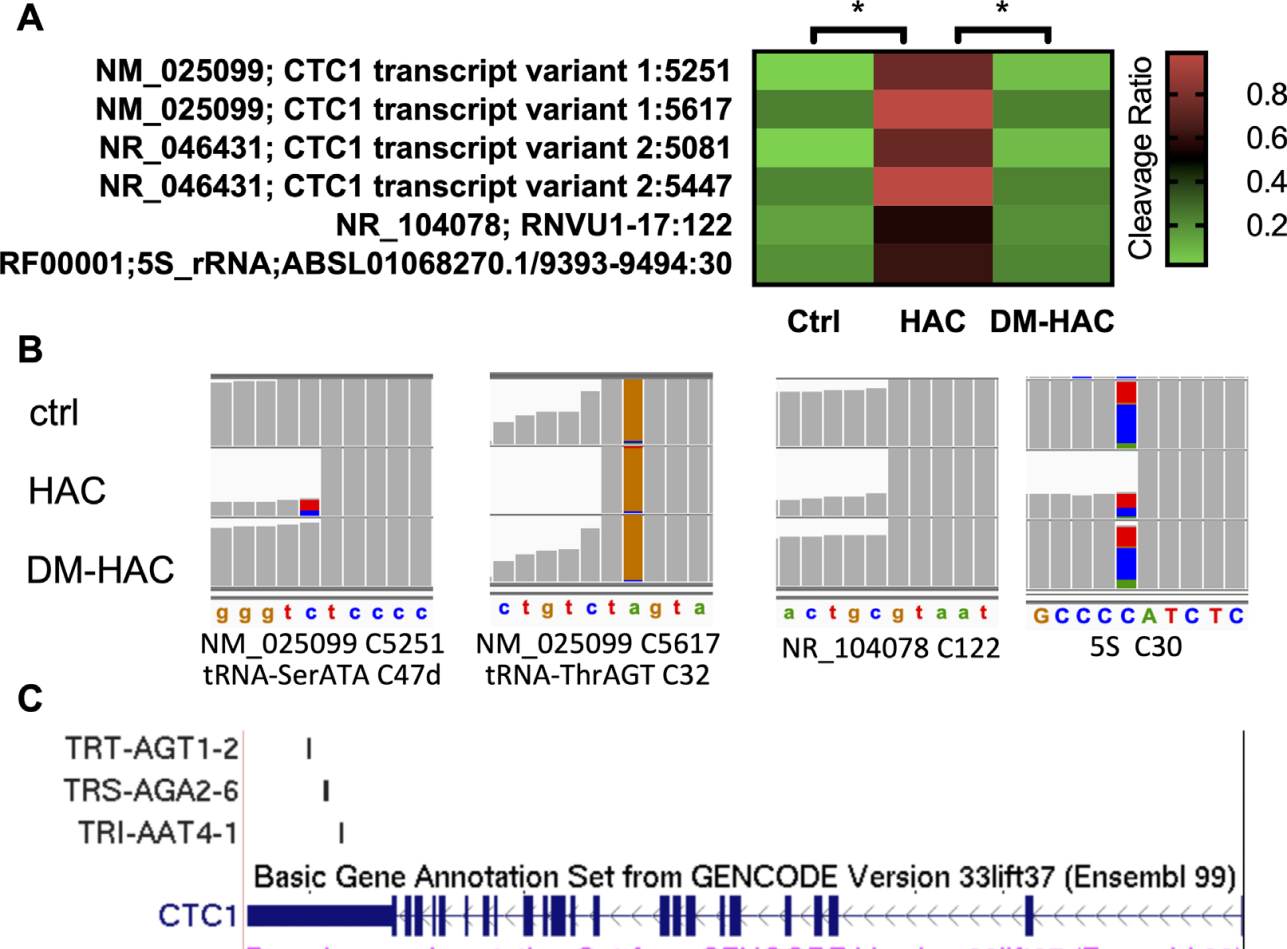
Transcriptome-wide analysis of m^3^C-modified RNA by HAC-seq. (**A**) Cleavage ratio heat map of m^3^C modification sites detected on mRNA and ncRNA species by HAC-seq with at least 50% cleavage in HAC samples. (**B**) Read alignments to NM_025999 at C5251 and C5617, NR_104078 at C12, and 5S rRNA at C30 in IGV using HAC-seq data. The location of the corresponding tRNA genes is also depicted. (**C**) A view of the *CTC1* locus from the UCSC Genome Browser. The 3′ untranslated region (UTR) of CTC1 contains three tRNA sequences: TRI-AAT4–1(tRNA IleAAT), TRS-AGA2–6 (tRNA SerAGA) and TRT-AGT1–2 (tRNA ThrAGT).

In summary, HAC-seq successfully identified a total of 48 m^3^C modification sites (Figures 3, 4 and Supplementary Table S1) corresponding to 17 different m^3^C sites on 11 cytoplasmic tRNA species and two mitochondrial tRNA species in MCF7 cells (Figure 6A). These m^3^C sites typically occur at a conserved sequence motif GGNCU (Figure 6B). All four tRNA-Ser isoacceptors (Ser-AGA, SerCGA, SerGCT, SerTGA) are modified at C32 of the A loop and/or C47d of the V loop. The other m^3^C-modified tRNA species contain one modification site at C32 (ArgCCT, ArgTCT, ThrAGT, ThrCGT, ThrTGT, mt-SerTGA, mt-ThrTGT) of the A loop, C47d (LeuCAG) of the V loop, or C20 (MetCAT) of the D loop (Figure 6A and C).

In addition to single-nucleotide resolution mapping of m^3^C sites on tRNA, HAC-seq is useful to estimate m^3^C modification levels on different tRNAs. Our LC-MS/MS and Northern blot analysis showed that the HAC conditions we used result in maximal RNA cleavage (Figure 1). AlkB treatment before the cDNA library preparation step largely decreased the RT-stop effects by m^3^C and other hard-stop modifications. Therefore, the cleavage ratio calculated from HAC-seq can be used to approximate the proportion of m^3^C modification at any individual site. However, for tRNA with two m^3^C sites, the cleavage ratio of the 5′ m^3^C site (e.g. m^3^C32 on tRNA-Ser) could be influenced by cleavage at the downstream m^3^C site (e.g. m^3^C47d on tRNA-Ser). Absolute m^3^C quantification for different tRNAs might however be possible by using appropriate standards (e.g. HAC-treated m^3^C-modified RNA oligos with known stoichiometry for calibration).

### Transcriptome-wide mapping of m^3^C modification by HAC-seq

Recent research has reported that m^3^C also exists on mRNA via LC-MS/MS analysis (26). Yet a comprehensive mapping of all m^3^C sites on mRNA as well as non-coding RNAs has not yet been reported. Based on the ability of HAC-seq to successfully identify m^3^C modification sites for tRNAs, we next mapped our HAC-seq data to Refseq mRNA dataset and Rfam ncRNA dataset. Cleavage ratios were calculated as described above. m^3^C modification level of mRNA is reportedly much lower than for tRNAs (26). Using the same HAC-seq data analysis pipeline developed for tRNA, we found that from amongst the tens of thousands of transcripts we sequenced, only 6 cleavage sites were considered m^3^C-positive on 4 different RNAs (Figure 6A and B). NM_025099 and NR_046431 are two transcript isoforms of CTC1 (CST Telomere Replication Complex Component 1) mRNA. Further investigation identified three tRNA genes at the *CTC1* locus that overlap with the 3′ UTR of CTC1 including m^3^C-modified tRNA-SerAGA and tRNA-ThrAGT (Figure 6C). Therefore these m^3^C sites most likely resulted from the cleavage of the overlapping tRNAs (tRNA-SerAGA m^3^C47d and tRNA-ThrAGT m^3^C32) but not the CTC1 mRNA (Figure 6B). If we lowered the cleavage-ratio cutoff for further RNA HAC-seq data analysis, more m^3^C-positive RNA candidates were identified. However additional validation is required to determine if these are true m^3^C sites rather than background sequencing artifacts. In conclusion, our transcriptome-wide analysis using HAC-seq identified very few other RNA species that contain comparable levels of m^3^C as tRNAs.

## DISCUSSION

The emergence of the Epitranscriptomics field over the past few years has been facilitated and stimulated by the rapid advancement in technologies for the widespread detection of individual RNA modifications. There is increasing evidence supporting the critical biological roles of different RNA modifications and their implication in various diseases (42). For example, dysregulation of some of the more well-studied RNA modifications including m^6^A is known to promote oncogenesis and the m^6^A MTase METTL3 has been identified as a possible new cancer therapeutic target (15). m^3^C is a conserved modification on tRNA from yeast to humans. Although m^3^C ‘writers’ and ‘erasers’ have been recently identified in mammalian cells (26,28,29), the molecular role of m^3^C is still poorly understood, in part due to the limitation of available methods for the specific and global detection of m^3^C modification throughout the transcriptome. In this report, we developed a chemical-based sequencing method, HAC-seq, to specifically profile the m^3^C methylome transcriptome-wide at single nucleotide resolution. HAC-seq is unbiased, high-throughput, and easy-to-perform. More importantly, HAC-seq is m^3^C-specifc and is able to estimate m^3^C modification levels on individual RNA species. Thus HAC-seq can be broadly applied to investigate the m^3^C methylome and facilitate research on the role m^3^C in normal physiology and diseases.

m^3^C is a hard-stop modification, meaning it arrests reverse transcription or induces misincorporations during cDNA synthesis. As a result, bioinformatic analysis of these RT stops and mismatches can map m^3^C and other hard-stop sites at the same time. Additionally, m^3^C can be removed by demethylation treatment with bacterial AlkB proteins. Three sequencing techniques were developed based on these features of m^3^C RNA modification—ARM-seq (41), HAMR (40) and DM-tRNA-seq (22,30). ARM-seq determines the m^3^C sites by comparing ratio of reads between AlkB-treated and untreated samples (41). HAMR determines the m^3^C sites by calculating the rate of mismatches (40). DM-tRNA-seq calculates a modification index which combines both RT stops and mismatch information without demethylase treatment (22,30). It further validates these modification sites by comparison with sequencing data with demethylase treatment (22,30). However all of these methods are highly dependent on the reverse transcription efficiency, and multiple other RNA modifications are hard-stop and AlkB-removable. Recently the AlkAniline-seq method was reported to simultaneously profile m^3^C and m^7^G modifications at single nucleotide resolution (31). AlkAniline-seq detected RNA modification sites by exposing RNA to alkaline conditions followed by aniline cleavage (31). However it remains unclear how this method detects m^3^C since HPLC-MS/MS from this publication shows that alkaline hydrolysis does not generate abasic sites at m^3^C but rather deaminates m^3^C to m^3^U which is not reactive to the subsequent Aniline-induced cleavage. Additionally, the detection of m^3^C site by AlkAniline-seq can be hindered by the cleavage at the downstream m^7^G site (31). While all of these techniques allow mapping of multiple modification types in a single sequencing dataset, this is also a limitation because the specific detection of m^3^C sites can be hampered by other modifications.

DM-tRNA-seq has been reported as a quantitative high-throughput sequencing approach by using a ‘Modification Index’ (MI) in their sequencing data under no-demethylase treatment condition (22). MI was calculated at each modification site by (number of mutations + stops at each position)/(total counts at each position) (22). Their data showed that almost all cytoplasmic tRNAs have m^1^A58 with MI of 0.8–1.0. Because of the high mutation/RT-stop rate at A58 which is downstream of a m^3^C modification site, the actual read coverage at m^3^C sites and quantification accuracy for m^3^C may be compromised in DM-tRNA-seq. In contrast, our HAC-seq data included demethylase treatment step before cDNA library preparation. Moreover we demonstrated that HAC cleaved RNA at m^3^C sites specifically. No chemical-induced cleavage sites were identified downstream of m^3^C. Therefore the cleavage ratio calculated from HAC-seq is able to more accurately estimate the m^3^C levels on RNA. DM-tRNA-seq using HEK293T and HeLa cells reported that m^3^C-high tRNAs include MetCAT-C20, LeuCAG-C47d, and SerTGA-C32 with MI of 0.8–1.0 (22). Our HAC-seq using MCF7 cells showed that there is some variability in m^3^C levels in different tRNA isoforms. Overall, cytoplasmic and mitochondrial tRNA-Thr species are the highest-m^3^C modified tRNAs with almost 100% cleavage at C32 by HAC treatment.

**Figure 7.**
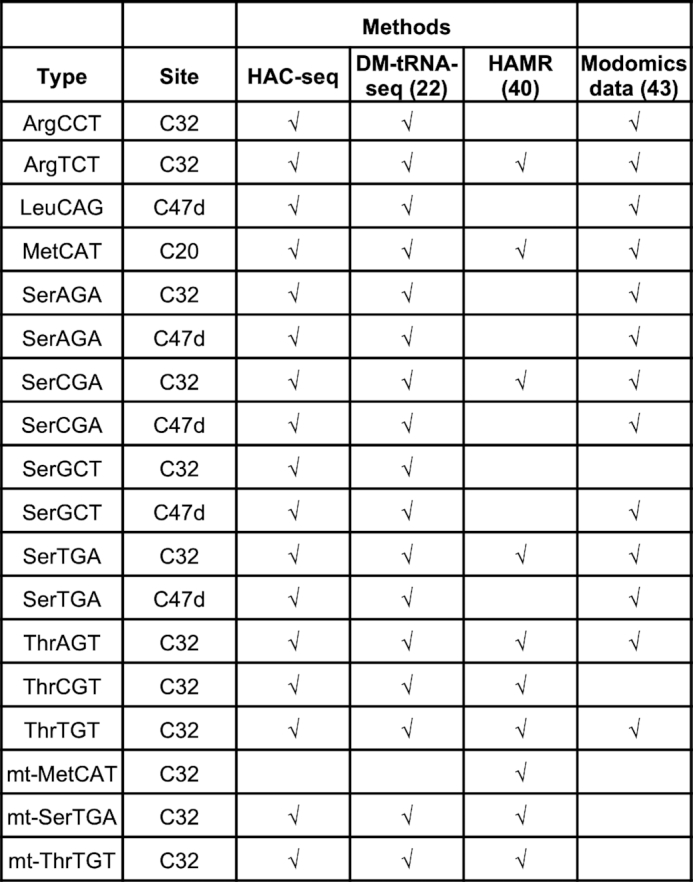
Comparison of tRNA m^3^C sites detected by HAC-seq and other methods. tRNA m^3^C methylomes reported by HAC-seq, DM-tRNA-seq, HAMR and Modomics dataset are summarized.

Our HAC-seq analysis identified 48 m^3^C modification sites corresponding to 17 different m^3^C sites on 11 cytoplasmic tRNA isoacceptors and 2 mitochondrial tRNA isoacceptors in MCF7 cells (Figure 5 and Supplementary Table S1). All m^3^C sites detected by HAC-seq and other methods including DM-tRNA-seq and HAMR are summarized in Figure 7 together with documented m^3^C modification sites from Modomics database (43). The cleavage ratios calculated from HAC-seq provide the first comprehensive view of the semi-quantitative stoichiometry of m^3^C modification of each modified RNA. LC-MS/MS is widely used to quantify the percentage of modifications on RNA. Xu *et al.* reported that m^3^C on tRNAs is 1000-fold greater than that on m^3^C on mRNA (26). However LC–MS/MS analysis cannot decipher the modification of individual RNAs and is not high-throughput. Using HAC-seq we found that tRNAs are the only m^3^C-modified RNA species with high stoichiometry. Almost no mRNA or non-coding RNAs were identified as m^3^C positive by HAC-seq. Similarly, no m^3^C signals were detected on polyA-enriched yeast mRNA or non-polyadenylated yeast non-coding RNA by AlkAniline-seq (31). This suggests that m^3^C modification is either limited to tRNAs or is present at very low stoichiometries for other RNA species that are below the limit of detection using stringent criteria for HAC-seq analysis. Future studies combining antibody-based enrichment of m^3^C-containing RNAs with HAC-seq will likely help resolve this question.

## DATA AVAILABILITY

HAC-seq data accession number: GSE157682.

## Supplementary Material

gkaa1186_Supplemental_Files
