# Supplementary material for: Nucleotide resolution profiling of m3C RNA modification by HAC-seq
Source: Nucleic Acids Res. 2020 Dec 11;49(5):e27. doi: 10.1093/nar/gkaa1186 (PMC7969016; doi:10.1093/nar/gkaa1186)
Supplement: gkaa1186_Supplemental_Files [file gkaa1186_supplemental_files.zip › JC revision NAR 101120 supplementary information.docx]

**Supplementary Figure 1. RNA mass spectrometry identification of false positive m^3^C sites detected by HAC-seq. (A)** Cleavage ratio plots of possible new m^3^C sites detected by HAC-seq. **(B)** LC-MS/MS analysis of m^3^C and m^5^C on isolated tRNA-ValCAC and tRNA-SerGCT. **(C)** IGV display of read alignments around the false positive m^3^C sites on tRNA-LeuCAG and tRNA-ValCAC.
